# Bilateral hyperkeratosis of the nipples and areolae with linear nevus and acanthosis nigricans: A rare case report

**DOI:** 10.1002/ski2.344

**Published:** 2024-01-27

**Authors:** Rui‐Ni Wang, Ping‐Shun Zhang, Xiao‐Fang Zhu

**Affiliations:** ^1^ Department of Dermatology of Dingbian County Hospital Shanxi China; ^2^ Department of Dermatology of Clinical Medical college of Yangzhou University Jiangsu China

## Abstract

Hyperkeratosis of the nipples and areolae (HNA) is an uncommon skin disorder with no definite aetiology. We report a case of a 16‐year‐old boy, who presented with bilateral pigmentation and thickening of the nipples and areolae, accompanied with linear brown protrusions on the anterior neck and a velvet like appearance with pigmentation on the axillary bilaterlly. Based on clinical and histopathological, and dermatoscopic findings, the diagnosis of bilateral HNA accompanied by linear nevus and acanthosis nigricans was made. The skin lesions were improved by treatment with topical calcipotriol gel.

## Graphical abstract

We report a case of 16‐year‐old boy, presented bilateral pigmentation and thickening of the nipples and areolae, accompanied with linear brown protrusions on the anterior neck and velvet like appearance with pigmentation on bilateral axillary. The skin lesions were improved by treatment with topical caspotriol gel.

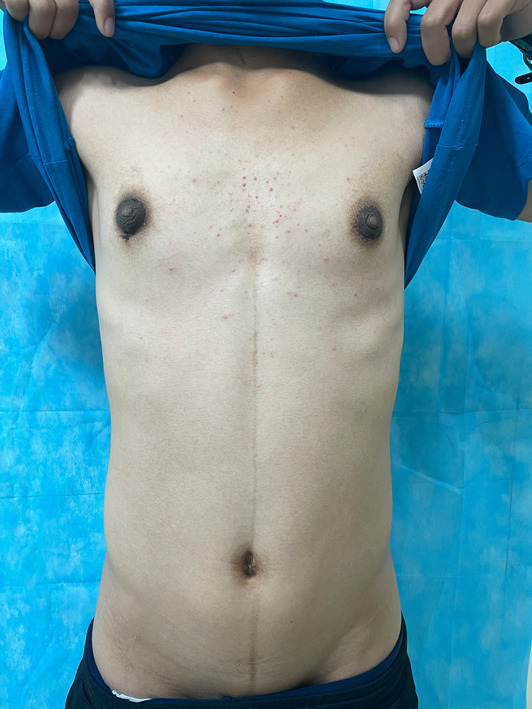

## 1 INTRODUCTION

Hyperkeratosis of the nipple and areola (HNA) is an infrequent dermatosis marked by the gradual verrucous thickening and brown pigmentation of the areola or nipple. This condition often coexists with various dermatoses, such as epidermal nevus, acanthosis nigricans, and mycosis fungoides. This case report outlines a rare occurrence of HNA accompanied by both a linear nevus and acanthosis nigricans.

## 2 CASE REPORT

**FIGURE 1:**
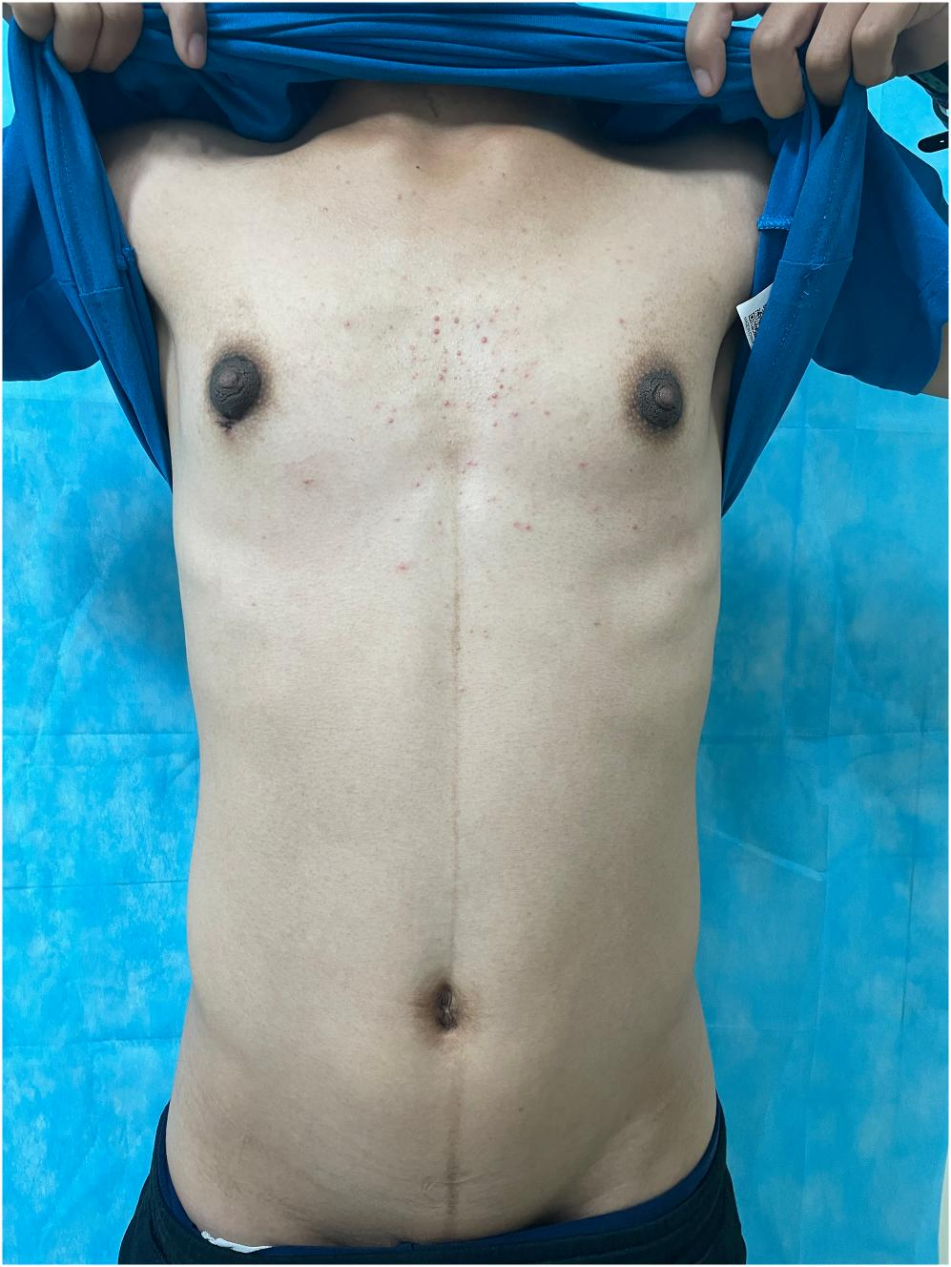
Clinical images of the patient: papillomatous thickening and black‐brown, papular, warty surfaces of the Bilateral nipples and areolae, and a dark brown pigmented line on the midline of the abdomen.

**FIGURE 2:**
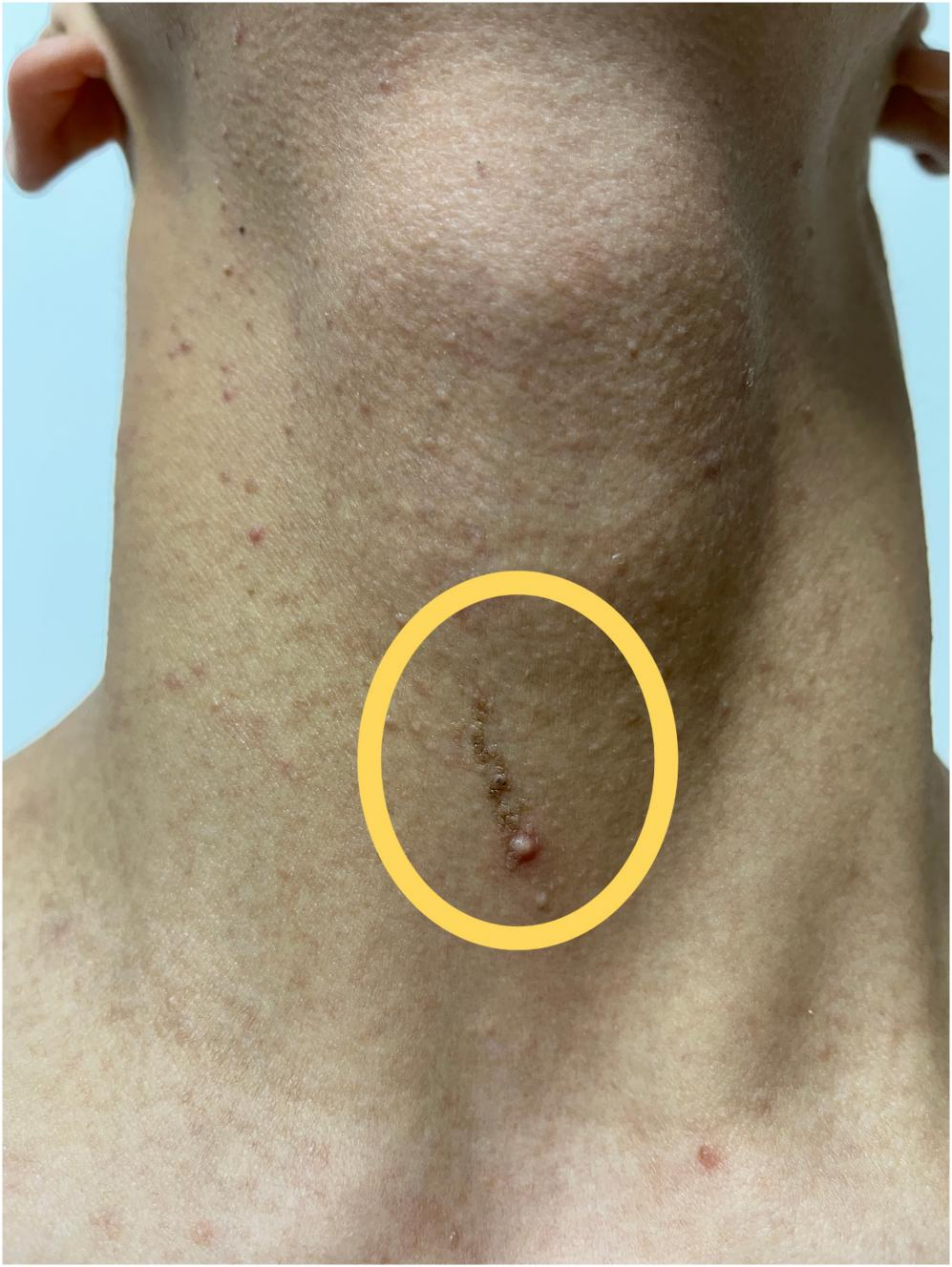
Linear brown protrusions on the anterior neck (as showed with yellow circle).

**FIGURE 3:**
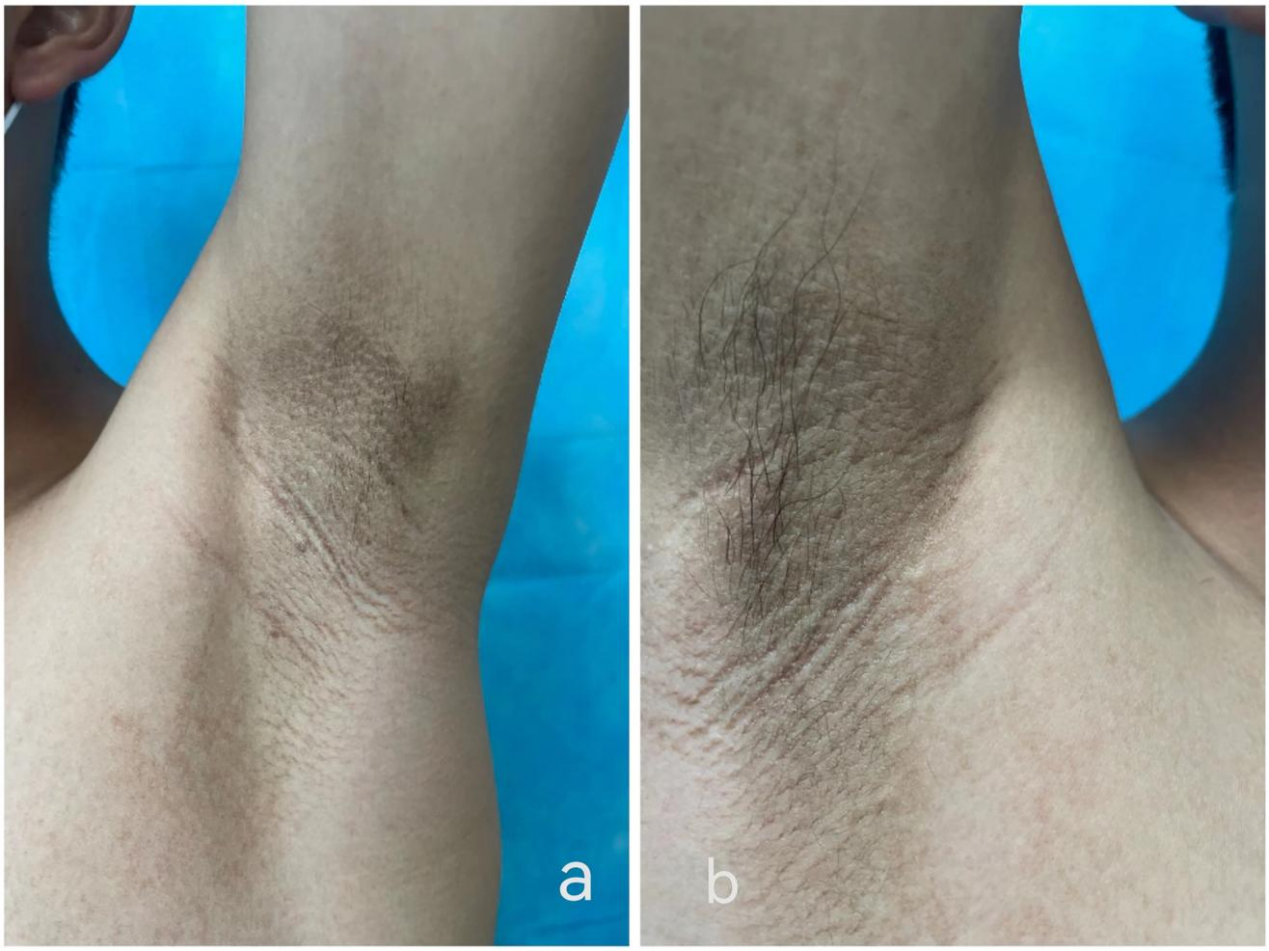
Slightly Velvet like appearance with pigmentation on right (a) and left (b) axillary region.

A 16‐year‐old boy presented to our dermatology department with a 1‐year history of bilateral pigmentation and thickening of the nipples and areolae. The gradual thickening of the areolae with a velvety appearance occurred a year ago with no apparent cause. The patient reported no itching or other discomfort, and the nipple colour was darker than the areola. There were no previous health issues, and no similar family history. Physical examination revealed papillomatous thickening and black‐brown, papular, warty surfaces of the nipples and areolae. Additionally, a dark brown pigmented line measuring over 10 cm in length was observed in the midline of the abdomen (Figure 1). Linear brown verrucous plaque measuring approximately 2 cm in length were visible on his anterior neck (Figure 2). A mild velvety appearance with pigmentation was observed in the bilateral axillary region (Figure 3a,b).

**FIGURE 4:**
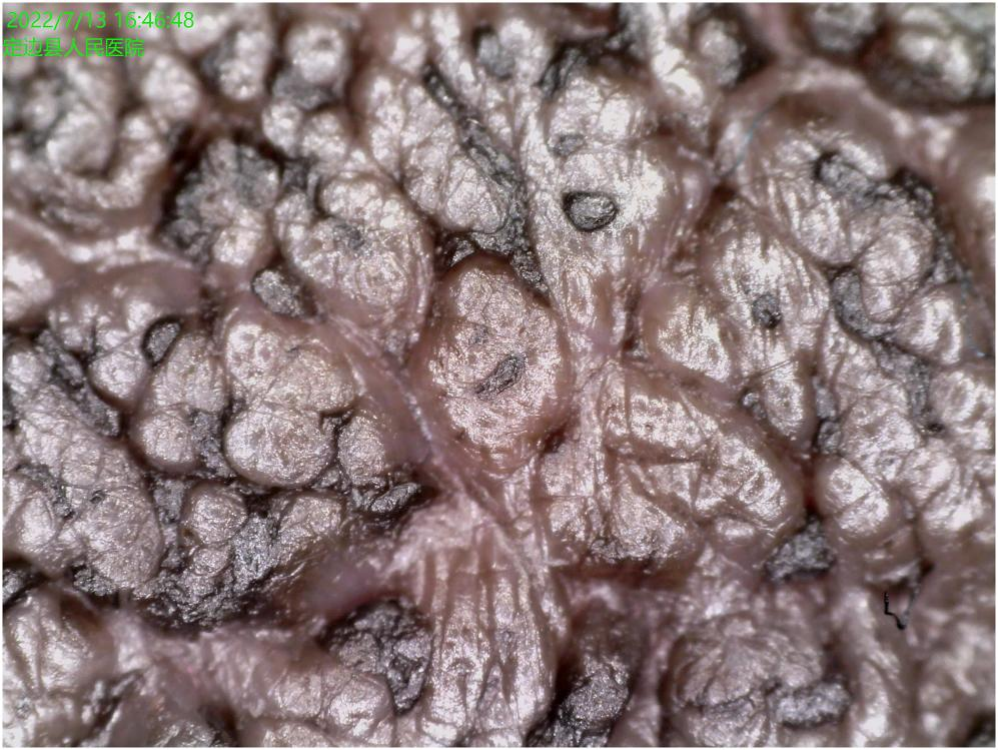
Dermoscopy showed cobblestone‐like structure with comedo openings.

**FIGURE 5:**
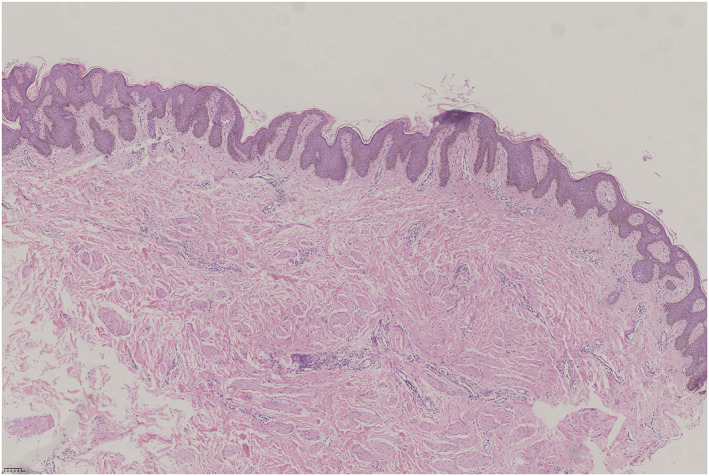
Histopathology showed slight hyperkeratosis and papillomatosis, epidermal basal layer hyperpigmentation and perivascular infiltration of mononuclear inflammatory cells in the dermis (haematoxylin and eosin stain; original magnification: ×20).

Laboratory examinations for thyroid function and endocrine hormone levels were normal. Dermoscopy of the areolae lesions revealed a cobblestone‐like structure with comedo openings (Figure 4). Pathological analysis of the left areola indicated mild hyperkeratosis and papillomatosis, increased pigmentation of the basal layer, perivascular infiltration of mononuclear inflammatory cells in the dermis, and chronic proliferative changes of collagen fibres in the dermis (Figure 5).

Based on clinical, histopathological, and dermatoscopic findings, the diagnosis of bilateral hyperkeratosis of the nipple and areola (HNA) was confirmed. Although the patient refused a skin biopsy of the axillary and anterior neck lesions, the clinical manifestations suggested a linear nevus on the anterior neck and acanthosis nigricans in the axillary region.

**FIGURE 6:**
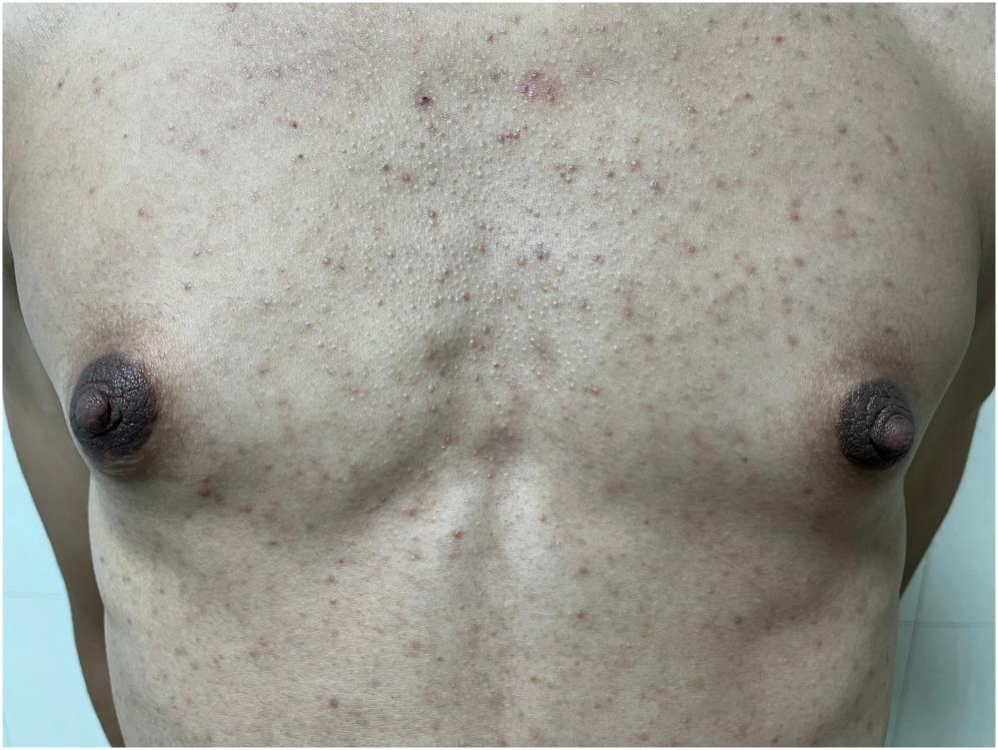
The patient's follow‐up photos showed slight improvement in the right nipples and areolae.

Considering the potential skin irritation caused by topical retinoids, the patient was prescribed topical 0.025% tretinoin cream and 0.005% calcipotriol ointment twice daily to the left and right nipples and areolas, respectively. After 3 months, improvement of the lesions on the right nipples and areolae was observed, but no improvement was seen on left the side. Therefore, the left nipple areolar rash was changed to topical 0.005% carpotriol ointment. However the patient discontinued the treatment due to a lack of further improvement (Figure 6).

## 3 DISCUSSION

HNA was initially documented by Tauber in 1923.^1^, ^2^ Formerly perceived as a rare, sporadic, and benign skin condition, there have been approximately 121 global reports on HNA,^3^ with ongoing increases in related literature. This suggests that the disease may not be as uncommon as previously believed, highlighting inadequacies in our prior comprehension, resulting in potential misdiagnoses. Although HNA is generally non‐threatening and lacks reported fatalities, it significantly affects patients' skin aesthetics and psychological well‐being. The disease, initially categorised into three types by Levy‐Frankel,^4^, ^5^ exhibits distinct clinical manifestations: Type I manifests as an expanded epidermal nevus, typically affecting a single nipple or areola; Type II is associated with other skin disorders such as ichthyosis, acanthosis nigricans, Darier's disease, chronic eczema, hereditary allergic dermatitis, and cutaneous T‐cell lymphoma, with lesions usually appearing bilaterally; Type III, congenital or naevoid, occurs independently of other dermatoses, mainly in women aged 20–30, involving usually both nipples or areolae.^6^ The reported patient presented with bilateral nipple and areola involvement, accompanied by acanthosis nigricans, placing the case within Type II. Intriguingly, the patient coincidentally exhibited a linear nevus, raising the possibility of classification as Type I according to Levy‐Frankel's system. Notably, both Type I and Type II are relatively rare compared to Type III. The aetiology of HNA remains unclear, with onset reported during puberty or pregnancy, sometimes resulting in complications during lactation post‐delivery.^7^ Male patients, particularly those treated with diethylstilboestrol for prostate cancer, have also been noted,^8^ suggesting a potential hormonal imbalance link. The relationship between HNA and benign acanthosis nigricans is uncertain, with some proposing an association with endocrine disorders. The presence of a dark brown pigmented line on the midline of the abdomen in the reported case may suggest an endocrine‐related cause. Histopathological similarities between nipple areolar hyperkeratosis and acanthosis nigricans have been observed. Wei's findings suggest localised immune system dysfunction and dysregulated keratin family expression in HNA pathogenesis.^2^ There is no specific treatment for this disease. Various therapeutic methods have been attempted, including keratolytics, topical corticosteroids, topical calcipotriol, topical retinoids, cryotherapy, CO2 laser, curettage, and radiofrequency ablation. Oral medications such as acitretin and glucocorticoids, as well as oral minocycline with anti‐inflammatory, immunomodulatory, and anti‐apoptotic properties, have been reported for HNA treatment.^9^ In the reported case, the skin lesions on the right side showed improvement following a 3‐month treatment with topical calcipotriol ointment, while the left side lesions did not respond to topical tretinoin cream.

Hyperkeratosis of the nipple and areola (HNA) is a rare dermatosis characterised by gradual verrucous thickening and brown pigmentation of the areola or nipple. This condition is often associated with various dermatoses, including epidermal nevus, acanthosis nigricans, mycosis fungoides, and others. In this particular case. HNA is exceptional as it coexists with both a linear nevus and acanthosis nigricans.

## CONFLICT OF INTEREST STATEMENT

None to declare.

## AUTHOR CONTRIBUTIONS

**Rui‐Ni Wang**: Writing—original draft (equal). **Ping‐Shun Zhang**: Data curation (equal). **Xiaofang Zhu**: Writing—review and editing (equal).

## ETHICS STATEMENT

Not applicable.

## Data Availability

Research data are not shared.
